# Antimycobacterial Assessment of Salicylanilide Benzoates including Multidrug-Resistant Tuberculosis Strains

**DOI:** 10.3390/molecules171112812

**Published:** 2012-10-31

**Authors:** Martin Krátký, Jarmila Vinšová, Jiřina Stolaříková

**Affiliations:** 1 Department of Inorganic and Organic Chemistry, Faculty of Pharmacy, Charles University, Heyrovského 1203, Hradec Králové 50005, Czech Republic; Email: martin.kratky@faf.cuni.cz; 2 Laboratory for Mycobacterial Diagnostics and Tuberculosis, Regional Institute of Public Health in Ostrava, Partyzánské náměstí 7, Ostrava 70200, Czech Republic; Email: Jirina.Stolarikova@zu.cz

**Keywords:** antimycobacterial activity, benzoic acid ester, cytotoxicity, *in vitro* activity, multidrug-resistant tuberculosis, salicylanilide, salicylanilide ester

## Abstract

The increasing emergence especially of drug-resistant tuberculosis has led to a strong demand for new anti-tuberculosis drugs. Eighteen salicylanilide benzoates were evaluated for their inhibition potential against *Mycobacterium tuberculosis*, *Mycobacterium avium* and two strains of *Mycobacterium kansasii*; minimum inhibitory concentration values ranged from 0.5 to 16 μmol/L. The most active esters underwent additional biological assays. Four benzoates inhibited effectively the growth of five multidrug-resistant strains and one extensively drug-resistant strain of *M. tuberculosis* at low concentrations (0.25–2 μmol/L) regardless of the resistance patterns. The highest rate of multidrug-resistant mycobacteria inhibition expressed 4-chloro-2-[4-(trifluoromethyl)-phenylcarbamoyl]phenyl benzoate (0.25–1 μmol/L). Unfortunately, the most potent esters were still considerably cytotoxic, although mostly less than their parent salicylanilides.

## 1. Introduction

Tuberculosis (TB) represents a contagious infectious disease caused by *Mycobacterium tuberculosis* complex. It is still a harsh global public health problem, partly due to increasing emergence of multidrug-resistant tuberculosis [MDR-TB, which was defined as the infection that is resistant to at least isoniazid (INH) and rifampicin (RIF), the most effective first-line oral agents], and most recently the extensively drug-resistant tuberculosis (XDR-TB). XDR-TB consists in MDR-TB in combination with both resistance to any fluoroquinolone and at least one second-line injectable drug (kanamycin, amikacin, capreomycin). Every year almost 500,000 people are infected with MDR-TB and about 40,000 new XDR-TB cases are appraised annually, with an increasing trend expected in the future. While the standard therapeutic regimen for drug-sensitive TB lasts six months, the treatment of MDR-TB usually takes at least 18 months, and XDR-TB is often untreatable; the coincidence with HIV infection brings other serious problem [1,2]. Therefore the development of novel antimycobacterial agents is still challenging, and new structures with innovative mechanisms of action are especially needed [1,3,4,5]. Moreover, infections caused by nontuberculous (atypical) mycobacteria bring some challenges including those in the area of new drug discovery. Compounds with collateral anti-TB and anti-nontuberculosis mycobacteria activity may bring a satisfactory progress [6].

Salicylanilide (2-hydroxy-*N*-phenylbenzamide) derivatives may be such a promising group with a complex mechanism of action [7]. Their various esters have exhibited a significant antimycobacterial activity in micromolar or lower concentrations, including MDR-TB and atypical mycobacteria; they do not share any resistance with established antimycobacterial drugs. Esterification of salicylanilides may bring some both pharmacodynamic and pharmacokinetic advantages [7,8,9,10,11,12].

Some substituted esters of benzoic acid (BA) with substituted phenols have displayed antimycobacterial properties against typical and atypical species [13,14]. It was reported that *M. tuberculosis* is uniquely susceptible to weak acids compared to other mycobacteria. Some ester prodrugs of benzoic acid expressed a significant activity, especially at slightly acidic environment [15]. Recently, four salicylanilide benzoates were reported to block the growth of drug-sensitive *M. tuberculosis* strain with minimum inhibitory concentrations (MICs) ranging between 0.5–2 µmol/L and, moreover, it was found that they act as mild inhibitors of isocitrate lyase and methionine aminopeptidase, two enzymes essential for the maintenance of mycobacterial infection. These targets are different from those affected by clinically used drugs [16]. Salicylanilide benzoates [2-(phenylcarbamoyl)phenyl benzoates; Table 1] were synthesized and their MICs against eight bacterial and fungal strains were reported [17].

This study brings a complex characteristic of antimycobacterial properties (including against atypical, MDR- and XDR-TB strains) of known salicylanilide benzoates. It is a part of our research effort concerned with a group of salicylanilide derivatives with improved activity and/or reduced toxicity in comparison to their parent molecules.

## 2. Results and Discussion

### 2.1. Chemistry

**Scheme 1:**
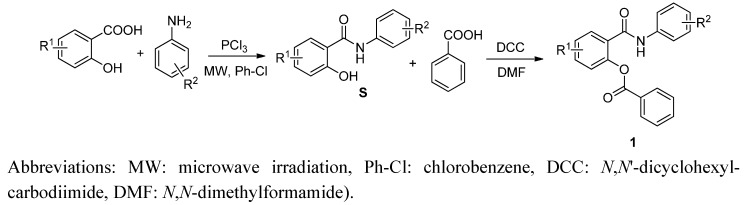
Synthesis of salicylanilides **S** and corresponding benzoates **1** (R^1^ for esters **1** = 4-Cl, 5-Cl, 4-Br; R^2^ = 3-Cl, 4-Cl, 3,4-diCl, 3-Br, 4-Br, 3-F, 4-F, 3-CF_3_, 4-CF_3_).

The synthesis and characterization (m.p., IR and NMR spectra, elemental analyses) of salicylanilide benzoates [2-(phenylcarbamoyl)phenyl benzoates; **1**] were published recently [17]; their synthetic route is depicted in Scheme 1. Yield of esters **1** ranged from 44 up to 88% [17].

### 2.2. *In Vitro* Antimycobacterial Evaluation

Eighteen salicylanilide benzoates were evaluated against four mycobacterial strains—one tuberculous and three atypical ones (*Mycobacterium avium* and two strains of *Mycobacterium kansasii*). Results are summarized in Table 1.

**Table 1:** Antimycobacterial activity of salicylanilide benzoates **1**.

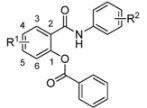													
			MIC [μmol/L]										
	R^1^	R^2^	*M. tuberculosis* 331/88		*M. avium* 330/88			*M. kansasii* 235/80			*M. kansasii* 6509/96		
			14 d	21 d	14 d		21 d	7 d	14 d	21 d	7 d	14 d	21 d
**1a**	4-Cl	3-Cl	2	2	**4**		8	**2**	4	8	4	4	8
**1b**	5-Cl	3-Cl	4	4	8		8	**2**	4	4	4	4	8
**1c**	4-Cl	4-Cl	4	4	8		16	4	8	8	4	8	8
**1d**	5-Cl	4-Cl	2	4	**4**		**4**	**2**	**2**	4	**2**	**2**	4
**1e**	4-Cl	3-Br	2	2	8		16	**2**	4	8	**2**	4	8
**1f**	5-Cl	3-Br	4	4	4		8	**2**	4	4	**2**	4	4
**1g**	4-Cl	4-Br	2	2	**4**		8	**2**	4	4	**2**	4	4
**1h**	5-Cl	4-Br	2	2	**4**		**4**	**2**	4	4	**2**	4	4
**1i**	4-Cl	3-F	4	4	8		16	**2**	4	8	**2**	4	4
**1j**	5-Cl	3-F	4	8	8		16	**2**	8	8	**2**	4	8
**1k**	4-Cl	4-F	4	4	8		16	**2**	8	8	**2**	4	4
**1l**	5-Cl	4-F	8	8	**4**		8	**2**	4	8	4	8	8
**1m**	4-Cl	3,4-diCl	**1**	**1**	8		8	**2**	4	4	**1**	**2**	**2**
**1n**	5-Cl	3,4-diCl	2	2	8		8	**1**	**2**	4	**1**	**2**	4
**1o**	4-Cl	4-CF_3_	**0.5**	**1**	**4**		**4**	**1**	**1**	**1**	**2**	**2**	**2**
**1p**	5-Cl	4-CF_3_	2	2	8		8	**1**	**2**	4	**1**	**2**	4
**1q**	4-Cl	3-CF_3_	2	2	8		16	**2**	4	4	**2**	4	4
**1r**	4-Br	4-CF_3_	**1**	**1**	**4**		**4**	**2**	**2**	**2**	**2**	**2**	**2**
**INH**	-	-	0.5-1	0.5-1	>250		>250	>250	>250	>250	2	4	4-8
**PAS**	-	-	62.5	62.5	32		125	125	1000	>1000	32	125	500
**BA**	-	-	>1000	>1000		>1000	>1000	1000	>1000	>1000	250	1000	1000

One or two best MIC values for each strain are given in bold; INH: isoniazid; PAS: *para*-aminosalicylic acid; BA: benzoic acid. MICs of **1m**, **1n**, **1o** and **1r** against *M. tuberculosis* 331/88 were taken from reference [16].

All tested compounds exhibited a significant activity against drug-sensitive *M. tuberculosis* at micromolar concentrations (0.5–8 μmol/L) with **1m**, **1o** and **1r** showing superiority (MICs ≤ 1 μmol/L). When we evaluated the isomers on the salicylic ring, 4-chloroderivatives were more beneficial than 5-chloro ones—only with **1c** *vs.***1d** being an exception and with no difference in the **1g** *vs.***1h** pair. The order of the moieties on the aniline ring is as follows (according to decreased potency): 4-CF_3_ ≥ 3,4-dichloro > 3-CF_3_ (limited data) ≈ 4-Br > 3-Cl = 3-Br > 4-Cl > 3-F > 4-F. The benzoylation of salicylanilides provided esters with noticeably improved activity when compared to the parent phenolic molecules **S** (Table 2) [18]—e.g., even eight-fold for **1m**; no ester exhibited inferior activity than its “parent” salicylanilide, and just **1b**, **1c**, and **1r** have identical MIC values.

**Table 2:** Antimycobacterial activity of parent salicylanilides **S** [18].

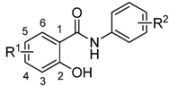								
			MIC [μmol/L]					
	R^1^	R^2^	*M. tuberculosis* 331/88		*M. avium* 330/88		*M. kansasii* 235/80	
			14 d	21 d	14 d	21 d	14 d	21 d
**S-a**	5-Cl	3-Cl	4	8	8	16	4	8
**S-b**	4-Cl	3-Cl	4	4	16	16	4	8
**S-c**	5-Cl	4-Cl	4	4	8	8	8	8
**S-d**	4-Cl	4-Cl	4	4	8	8	4	8
**S-e**	5-Cl	3-Br	NT	NT	NT	NT	NT	NT
**S-f**	4-Cl	3-Br	NT	NT	NT	NT	NT	NT
**S-g**	5-Cl	4-Br	8	16	8	8	4	4
**S-h**	4-Cl	4-Br	4	4	16	16	4	4
**S-i**	5-Cl	3-F	8	8	31	31	8	8
**S-j**	4-Cl	3-F	8	16	32	32	32	32
**S-k**	5-Cl	4-F	16	16	16	16	4	4
**S-l**	4-Cl	4-F	16	16	32	32	16	32
**S-m**	5-Cl	3,4-diCl	4	8	16	16	4	4
**S-n**	4-Cl	3,4-diCl	4	4	16	16	8	8
**S-o**	5-Cl	4-CF_3_	2	2	8	8	1	1
**S-p**	4-Cl	4-CF_3_	4	4	8	8	4	4
**S-q**	5-Cl	3-CF_3_	NT	NT	NT	NT	NT	NT
**S-r**	4-Br	4-CF_3_	1	1	1	1	2	4

NT: not tested. MICs of salicylanilides **S** were taken from reference [18].

*M. avium* showed the lowest level of susceptibility among the investigated mycobacterial strains (MICs 4–16 μmol/L). Compounds **1d**, **1h**, **1o** and **1r** are the most active esters. In general, derivatives substituted in the aniline part by 3-chloro (**1a**, **1b**), 4-bromo (**1g**, **1h**), and 4-trifluoromethyl (**1o**, **1p**, **1r**) moieties exhibited better activity; on the other hand, 3-fluoroderivatives (**1i**, **1j**) offered minimal benefit. With two exceptions, molecules derived from 5-chlorosalicylic acid showed a higher or equal activity than their 4-chloro isomers. The introduction of a benzoyl fragment into salicylanilide molecules resulted in an increased activity against *M. avium*—only three MIC values are higher than those of the parent salicylanilides (Table 2) [18], while others are equal or mostly lower in the case of benzoates, even four times in some cases.

Both clinically isolated and collection strains of *M. kansasii* were inhibited by salicylanilide benzoates **1** with MICs ≤ 8 μmol/L with clear **1o** superiority. 4-CF_3_, 3-CF_3_, 3,4-diCl and 4-Br represent the more suitable aniline substitution patterns; no substituent of the aniline part was evaluated as being significantly less beneficial than others. The influence of the halogen position on the salicylic ring is ambiguous. When concentrated on MICs towards the strain 235/80, there is a surprising fact—when 5-chloro-2-hydroxy-*N*-phenylbenzamides are esterified, the activity against *M. kansasii* did not change (or was even diminished for **1k** *vs.***S-k**), whereas masking of the phenolic group of 4-chloro-2-hydroxy-*N*-phenylbenzamides resulted mostly in improved *in vitro* activity (up to four times); only **1h** retained the same MIC values. Under our experimental conditions, benzoic acid itself revealed no activity against *M. tuberculosis* and *M. avium* and only a very weak inhibition potency for *M. kansasii* (MICs ≥ 250 μmol/L).

The most active derivatives proved a similar efficacy when compared to INH against drug-sensitive *M. tuberculosis*, and all derivatives exhibited substantially higher activity against *M. avium* and *M. kansasii* 235/80; the activities of INH and benzoates **1** against *M. kansasii* 6509/96 are almost identical. Second-line oral drug PAS seems to be significantly less active than the newly synthesized derivatives against all tested strains.

Salicylanilide benzoates expressed predominantly lower or equal MIC values in comparison to corresponding acetates [8] and benzenesulfonates [12]. Carbamates possessed a slightly higher *in vitro* inhibitory activity for *M. tuberculosis*, whereas MIC levels against atypical strains are approximately similar [9]. Salicylanilide *N*-acetyl-L-phenylalanine esters demonstrated a superior activity against *M. tuberculosis* and somewhat worse against *M. avium* [10]. Benzoates surpassed the antimycobacterial activity of salicylanilides esters with different *N*-benzyloxycarbonyl α-amino acids [11].

In conclusion, the benzoylation of salicylanilides **S** led to derivatives with predominantly higher *in vitro* activity against all four mycobacterial strains. The aim of improving the antimycobacterial potency was successfully achieved. The reason may lay in the increased lipophilicity of synthesized esters, which facilitates passage through biomembranes. With respect to the weak intrinsic activity of benzoic acid against *M. kansasii*, the possibility of synergistic action of released salicylanilides and benzoic acid could be included, as it has been previously observed.

### 2.3. *In Vitro* Activity against Drug-Resistant Tuberculosis Strains

Four esters with the lowest MICs (≤1 μmol/L against any mycobacterial strain) were selected for advanced biological tests. Benzoates **1m**, **1n**, **1o** and **1r** were evaluated for their *in vitro* activity against five MDR-TB strains and one XDR-TB strain (Table 3). All four derivatives exhibited very low MICs (0.25–2 μmol/L). Interestingly, in most cases MDR strains are even more sensitive than drug-sensitive *M. tuberculosis*. This susceptibility is independent on the resistance patterns indicating no cross-resistance with the conventionally used drugs.

**Table 3:** Activity of selected benzoates against multidrug-resistant strains.

	MIC [μmol/L]												
		*M. tuberculosis* 234/2005		*M. tuberculosis* 53/2009		*M. tuberculosis* Praha 1		*M. tuberculosis* Praha 131		*M. tuberculosis* 7357/1998		*M. tuberculosis* 9449/2006	
		14 d	21 d	14 d	21 d	14 d	21 d	14 d	21 d	14 d	21 d	14 d	21 d
**1m**		**0.25**	0.5	**0.5**	1	**0.5**	**0.5**	0.5	1	0.5	0.5	**0.5**	1
**1n**		**0.25**	0.5	1	2	1	1	0.5	1	0.5	1	2	2
**1o**		**0.25**	**0.25**	**0.5**	1	**0.5**	**0.5**	**0.25**	0.5	**0.25**	0.5	**0.5**	**0.5**
**1r**		0.5	0.5	1	2	1	1	0.5	1	**0.25**	0.5	1	1
**INH**		14.6	14.6	14.6	14.6	14.6	14.6	14.6	14.6	14.6	14.6	58.3	58.3

The best MIC value for each strain is given in bold; INH: isoniazid. MDR-TB strains: 357/2005 and 7357/1998 (both resistant to INH, RIF, rifabutine, streptomycin, ethambutol and ofloxacin); 53/2009 (resistant to INH, RIF, rifabutine, streptomycin, ethambutol); Praha 1 (resistant to INH, RIF, rifabutine, streptomycin, ethambutol and clofazimine) and 9449/2006 (resistant to INH, RIF, rifabutine and streptomycin); XDR-TB strain: Praha 131 (resistant to INH, RIF, rifabutine, streptomycin, ethambutol, ofloxacin, gentamicin and amikacin).

4-Trifluoromethyl derivative **1o** was assayed as the most active compound. Based on the pair **1m** *vs.* **1n**, the preferable location of the chlorine is the position 4 of the salicylic ring (compound **1m**). Compound **1o**, the derivative of 5-chlorosalicylic acid, exhibited a better *in vitro* activity than **1r**, which was synthesized from 5-bromosalicylic acid. The salicylanilide benzoates **1m**, **1n**, **1o**, and **1r** exhibited a higher activity against drug-resistant strains (expressed as MICs) than salicylanilide esters with *N*-acetyl-L-phenylalanine [10] and similar or slightly better MIC values when compared to salicylanilide carbamates [9].

### 2.4. Cytotoxicity Evaluation

Salicylanilides and their esters were referred to share some toxic effects on eukaryotic cells [7,8,9,10,16,19]. Therefore we examined the *in vitro* cytotoxicity of three most anti-MDR-TB active benzoates (compounds **1m**, **1o**, **1r**) and their parent salicylanilides (**S-m**, **S-o**, **S**-**r**) in the liver Hep G2 model. The cytotoxicity values, which are expressed as IC_50_, *i.e.*, concentration which decreases the viability of the cells to 50% from the maximal viability, was taken over from reference [16] (Table 4).

**Table 4:** Cytotoxicity and selectivity indexes of selected salicylanilides **S** and their benzoates **1**.

	IC_50_ [µmol/L] Hep G2	SI for *M. tuberculosis* 331/88		SI for MDR-TB strains		SI for XDR-TB strain	
		14 d	21 d	14 d	21 d	14 d	14 d
**2m**	2.54	2.54	2.54	5.08–10.16	2.54–5.08	5.08	2.54
**2o**	2.40	4.80	2.40	4.80–9.60	2.40–9.60	9.60	4.80
**2r**	2.34	2.34	2.34	2.34–9.36	1.17–4.68	4.68	2.34
**S-m**	0.84	0.21	0.10	-	-	-	-
**S-o**	0.36	0.18	0.18	-	-	-	-
**S-r**	2.71	2.71	2.71	-	-	-	-

IC_50_ and MIC values of salicylanilides against *M. tuberculosis* were taken from reference [16]. SI = IC_50_/MIC_100_.

The esterification of 5-chloro-*N*-(3,4-dichlorophenyl)-2-hydroxybenzamide (**S-m**) and 5-chloro-2-hydroxy-*N*-[4-(trifluoromethyl)phenyl]benzamide (**S-o**) by benzoic acid led to the derivatives with significantly decreased toxicity (approximately three and seven times, respectively); contrarily, the benzoylation of **S-r** has resulted in a slightly higher cytotoxicity (2.71 *vs.* 2.34 µmol/L).

Based on the comparison of MIC and IC_50_, it is possible to predict that the antimycobacterial activity of salicylanilide benzoates is not only the result of a general cytotoxic impact, but that they probably should have additional specific effect(s) against *M. tuberculosis*—e.g., recently reported inhibition of isocitrate lyase and methionine aminopeptidase [16].

However, benzoates still exhibited IC_50_ values in micromolar range similar to the activities against atypical mycobacteria. Selectivity indexes (SI) for *M. tuberculosis* ranges from 2.34 to 4.80. The situation for MDR-TB and XDR-TB strains is quite advantageous with SI values of 1.17–10.16; the ratios are more favourable for **1o**. Generally, only SI values about the break point of 10 could be considered to be still border sufficient, others are poor.

Although benzoic acid possesses only a very mild cytotoxicity (IC_50_ of 2,881 µmol/L in our assay), unfortunately the benzoylation did not fill up our expectation about the significant toxicity reduction of parent salicylanilides, in contrast to improved antimycobacterial efficacy. Otherwise, esterification of salicylanilides may be a perspective way to reduce undesired cytotoxicity; it is necessary to search new acids, because benzoic acid brings a certain, but insufficient benefit.

## 3. Experimental

### 3.1. Chemistry

Synthesis and characterization of the presented 2-(phenylcarbamoyl)phenyl benzoates **1a–r** was published by Krátký *et al*. [17].

### 3.2. *In Vitro* Antimycobacterial Susceptibility Testing

All compounds were tested against *Mycobacterium tuberculosis* 331/88 (H_37_Rv) (dilution of the strain was 10^−3^) and three nontuberculous strains: *Mycobacterium avium* 330/88 (resistant to INH, RIF, ofloxacin and ethambutol; dilution 10^−5^) and two strains of *Mycobacterium kansasii*—235/80 (dilution 10^−4^) and clinically isolated strain 6509/96 (dilution 10^−5^). The description of the used method can be found in [12]. The following concentrations of esters were used: 1,000, 500, 250, 125, 62.5, 32, 16, 8, 4, 2, 1, 0.5, 0.25 and 0.125 µmol/L. MIC (µmol/L) is the lowest concentration at which the complete inhibition of mycobacterial growth was occurred. Isoniazid (INH) and structurally similar *para*-aminosalicylic acid (PAS) were chosen as reference drugs for the comparison. The most active compounds were evaluated in the similar conditions and concentrations against six MDR-TB strains (dilution 10^−3^) with different resistance patterns: 7357/1998, 234/2005, 53/2009, 9449/2006, Praha 1 and Praha 131 (XDR-TB strain). All these strains were resistant to INH, rifamycines, and streptomycin; in some cases additional resistance was present.

## 4. Conclusions

In summary, salicylanilide benzoates revealed a significant antimycobacterial activity; their mechanism of action is still not fully elucidated and seems to be multiple. The masking of salicylanilide phenolic group by lipophilic aromatic acid resulted in the derivatives with improved antimycobacterial potency in the micromolar range (0.25–16 μmol/L). Additionally, the most active esters stopped the growth of MDR-TB strains with MIC values from 0.25 μmol/L. Salicylanilide benzoates represent a group with a promising *in vitro* antimycobacterial activity. Nevertheless, the expectancy of the reduced cytotoxicity was accomplished only partly—two esters of three tested ones exhibited a significantly lower toxicity when compared to parent salicylanilides, but these molecules are unfortunately still relatively toxic. Thus, the next search for new highly active and less cytotoxic derivatives still remains a topic of interest.
